# Short- and Medium-Term Outcomes Assessment of Urethral Prostatic Lift (UroLift) as a Minimally Invasive Treatment for Benign Prostatic Hyperplasia in a Tertiary Care Centre

**DOI:** 10.7759/cureus.75579

**Published:** 2024-12-12

**Authors:** Anna Akpala, Tamara Lezama, Mushtaq Hussain, Kehinde Jinadu, Tia Parker, Sahana Sreenivasan, Amar Manandhar

**Affiliations:** 1 Urology, Queen Elizabeth Hospital Birmingham, Birmingham, GBR; 2 Cardiac Surgery, Birmingham Women's and Children's NHS Foundation Trust, Birmingham, GBR; 3 Urology, University Hospitals of Birmingham, Birmingham, GBR

**Keywords:** benign prostatic hyperplasia (bph), enlarged prostate, lower urinary tract symptoms (luts), transurethral resection of the prostate (turp), urolift

## Abstract

Introduction and aim: Benign prostatic hyperplasia (BPH) is the enlargement and overgrowth of the prostate leading to the compression of the urethra and resulting in obstruction to the outflow of urine. Prostatic urethral lift (UroLift) is a budding minimally invasive technique that utilises mechanical manipulation of the prostate tissue so that the urethra is free from compression, thereby creating a channel for the outflow of urine. The aim of the audit was to assess the short- to medium-term outcomes in our centre in terms of improvement in symptoms, quality of life (QoL) and complication rates.

Method: A retrospective observational study was employed. All patients who had UroLift procedure between December 2021 and December 2022 were included.

Results: Sixty-four patients were found for the chosen period. Age ranged between 48 and 91 with a mean age of 73. The average prostate size was 48.63 g (31-50 g). Of the patients, 64% (n=41) had the procedure performed under general anaesthesia, whilst the rest had either regional or local anaesthesia or entonox. Two to eight clips were used per patient, with four being the median number of clips. Furthermore, 53.13% (n=34) had a catheter post-procedure, with 52.94% (n=18) of these achieving trial without catheter (TWOC) in less than seven days. Of the patients, 67.18% (n=43) had no complications, 7.8% (n=5) went into acute urinary retention requiring short-term catheterisation, 1.56% (n=1) had bleeding requiring blood transfusion and 6.25% (n=4) had infection. Overall, 17.18% (n=11) had failure of procedure requiring other forms of intervention for BPH at two years post-operatively. The median improvement in post-void residual scan and quality of life (QoL) scores were 58 mL and 1.5, respectively.

Conclusion: Although transurethral resection of the prostate (TURP) remains the gold standard surgical treatment for BPH, UroLift offers a less minimally invasive approach with better short-term recovery, preservation of sexual function and low complication profile. The failure rate from UroLift from previous studies falls within the range of 7%-22%, and the figures attained in our centre are comparable to these.

## Introduction

Benign prostatic hyperplasia (BPH) is a condition in which the prostate gland becomes enlarged due to an overgrowth of prostate tissue. Histologically, it is distinguished by non-malignant hyperplasia brought on by the proliferation of stromal and epithelial cells in the prostate's transition zone [1]. This hyperplasia causes a benign enlargement of the glandular tissue surrounding the prostatic urethra, pushing against the urethra and the bladder, thereby blocking the flow of urine and subsequently causing lower urinary tract symptoms (LUTS) [1].

The prevalence of BPH increases with age with 8%, 50% and 80% in the fourth, sixth and ninth decades of life, respectively [2]. The symptoms may include incomplete emptying of the bladder, urinary retention, frequency, urgency, incontinence, sexual dysfunction, nocturia and urinary tract infections (UTIs) [3].

Treatment for BPH depends on the severity of the patient's symptoms, fitness for surgery and patient's choice. Management options include conservative management/lifestyle changes, medical therapy and surgical management. Medical therapy involves the use of alpha-blockers and 5-alpha reductase inhibitors such as tamsulosin and finasteride. Surgical management includes minimally invasive procedures, Rezum steam ablation, photo-selective vaporisation of the prostate ("green light" laser prostatectomy) and prostatic urethral lift (UroLift). Others include transurethral resection of the prostate (TURP), currently the gold standard for managing BPH, holmium laser enucleation of the prostate (HoLEP) and prostatic artery embolisation.

The aim of this article is to assess the outcomes and complications of UroLift at our facility and compare them to national guidelines and other published data to ensure our patients are benefitting maximally from the procedure.

UroLift is a promising minimally invasive therapeutic approach for the treatment of benign prostatic hyperplasia. To remove the obstructing tissue from the urethra, a UroLift operation entails putting a telescope into the urethra and placing two to four implants into the prostate. The implants are made with common implantable materials: nitinol, stainless steel and polyethylene terephthalate (PET) suture. It is intended to increase urine flow without causing any prostate tissue to be burned or removed [4]. The implants are placed between the inner and outer surfaces of the prostate to pull the obstructing prostate lobes away from the urethra. Usually, after three months, they integrate into the prostate tissue and are no longer visible in the bladder [3,4].

UroLift has very strict inclusion requirements, and the procedure's long-term effectiveness depends on cautious patient selection. The inclusion criteria as documented by Jones et al. state to include men who are over 50 years of age, with a prostate volume of 20-70 mL on ultrasonography, International Prostate Symptom Score (IPSS) > 12, Qmax < 15 mL/s and a post-void residual volume < 350 mL [4]. The recommendation by the National Institute for Health and Care Excellence (NICE) is similar, stating that UroLift be performed as a day case or outpatient procedure as an alternative to TURP and HoLEP in people aged 50 and older with a prostate volume of 30-80 mL [5].

In comparison to other surgical options, UroLift is fast becoming a preferred option due to its favourable surgical outcomes, decreased recovery time, marked improvement in quality of life (QoL), reduction in LUTS and diminished sexual side effects such as retrograde ejaculation [3].

The majority of UroLift's side effects are transient and usually self-limited. These include burning and stinging when passing urine, haematuria, pain or discomfort, reoccurrence of prior symptoms, urgency, urge incontinence, retention, temporary urethral catheterisation, infection and anaesthetic risk, most of which tend to settle within a week [6].

## Materials and methods

This was a retrospective observational study to include all patients who had UroLift procedure for BPH in one National Health Service (NHS) trust between December 2021 and December 2022 with follow-up till date. Two patients who had previous surgical management for BPH were excluded. The data was obtained from patient's electronic records following institutional approval from the clinical audit and registration and management system of the hospital, and a total of 64 patients were found who fit the criteria. Following failed medical management of BPH (defined as patients with persisting LUTS due to BPH with no response to medical treatment in the form of alpha antagonist or 5-alpha reductase inhibitors), patients referred for UroLift were selected according to NICE guidelines, which recommend UroLift for men > 50 years of age with prostate volumes of 30-80 mL. The data collected included age, prostate size, the International Prostate Symptom Score (IPSS) and the quality of life score (QoL), flexible cystoscopy findings, post-void residual scan, number of clips used, type of anaesthetic, number of days until trial without catheter (TWOC), post-operative complications and readmission rate. Pre-operatively, all patients were evaluated for LUTS from history-taking, physical examination, ultrasound scan and estimation of prostate size, uroflowmetry and post-void residual scan. Symptom severity was assessed using the IPSS and the quality of life (QoL) score to assess the impact of their symptoms on their quality of life. All patients underwent flexible cystoscopy to assess the anatomy of the prostate and suitability for the procedure.

All patients were planned as a day case procedure, and all patients were administered local anaesthetic in the form of 2% lignocaine gel per urethra. Some patients were converted to general anaesthesia when they were unable to tolerate the procedure under local anaesthetic with or without sedation, whilst a few patients were planned for general anaesthetic ab initio. Patients received prophylactic intravenous amoxicillin 1 g and gentamicin 3 mg/kg as antibiotic cover for the procedure if no allergies.

The number and location of clips used were chosen at the discretion of the surgeon, as well as the insertion of urethral catheter post-procedure. The results were entered into an Excel sheet (Microsoft Corp., Redmond, WA), and following a quality assurance review, statistical analysis was performed using the Kruskal-Wallis test. The value at which p is considered statistically significant is set at p<0.05.

## Results

Age

The age range in the data set was 48-91, with a mean and median age of 73. Only one patient was below the cut-off age of 50 as recommended by NICE for consideration of UroLift.

Peri-operative measurements

The peri-operative measurements are listed in Table 1.

**Table 1:** Peri-operative parameters The data is represented as number and %. IPPS: International Prostate Symptom Score, QoL: quality of life, TWOC: trial without catheter, PVR: post-void residual

Parameter	Value
Median prostate size (g)	40
Median IPSS score (number)	28
Median Qmax (mL/s)	8.5
Median PVR (mL)	150
Median QoL score (number)	5
Local anaesthesia (number (%))	8 (12.5%)
General anaesthesia (number (%))	41 (64.06%)
Entonox (n (%))	12 (18.75%)
Regional anaesthesia (number (%))	3 (4.69%)
Median clips deployed (number)	4
Day case (number (%))	46 (71.87%)
Median time to TWOC (days)	5

Prostate size

In our study, 50% (n=32) of the patients had a prostate size between 31 and 50 mL, 12.5% (n=8) had a prostate size of less than 30 mL, 35.93% (n=23) had a prostate size between 51 and 80 mL and only one patient had a prostate size > 80 mL. The mean prostate size was 48.63 mL. Using the Kruskal-Wallis test, we found no correlation between prostate size and the number of clips used (p=0.0616).

Flexible cystoscopy findings

The flexible cystoscopy findings revealed that 82.81% (n=53) had no median lobe enlargement, 10.93% (n=7) were reported as bilobar occlusive prostate with small median lobe and 6.25% (n=4) had trilobar occlusive prostate.

Number of clips used

A total of four clips were used in 48.43% (n=31) of patients and six clips in 21.88% (n=14) of patients. The highest number of clips used was eight, and this was in one patient only, with two being the lowest number of clips used in five (7.81%) patients. The mean number of clips used was 4.5, and the median number was 4.

Anaesthesia use

Of the patients, 64.06% (n=41) had the procedure under general anaesthetic, whilst 12.5% (n=8) was performed under local anaesthetic. Using Fisher's exact test, there was no statistically significant association between the anaesthetic used and complications (p=0.8055). Furthermore, there was no association between hospital stay (day case rate) and anaesthetic use (p=0.1174).

Post-operative catheter and TWOC

At the end of the procedure, 53.12% (n=34) of the patients had a temporary catheter inserted. Furthermore, 35.29% (n=12) had a successful TWOC at 3-7 days post-operatively, 29.41% (n=10) at 8-14 days post-operatively and 14.7% (n=5) in less than one day post-operatively. Seven (20.59%) patients unfortunately failed TWOC. The median time to TWOC is five days post-operatively.

Complications

Well-documented complications following the UroLift procedure include bleeding, pain, discomfort and dysuria. Serious complications in this study are complications experienced by patients requiring any form of intervention beyond reassurance and have been highlighted in Table 2.

**Table 2:** Complications following UroLift The data is represented as number and %.

Complication	Number	%
No complication	43	67.18%
Urinary retention	5	7.81%
Infection	4	6.25%
Bleeding requiring transfusion	1	1.56%
Failure of procedure	11	18.75%

Readmission rate

Out of the 64 patients, two (3.13%) required readmission, one for urinary retention and the other for catheter-related issues.

Failure rate

At two-year post-operatively, a total of 11 patients had a failure of procedure, out of which 63.63% (n=7) required other forms of surgical management in the form of TURP. Four patients are on long-term catheter, and one patient is performing clean intermittent self-catheterisation for the management of LUTS.

Successful outcome

In the successful patients, we compared their pre-operative parameters with their post-operative parameters and found an improvement in the quality of life scores from a pre-operative median of 5 to a post-operative median of 3.5, indicating a 1.5 positive difference. The median IPPS pre-operatively was 28 (which is within the severely symptomatic range), and the median IPPS was 15 post-operatively, indicating a significant improvement in their symptoms. The mean pre-operative post-void residual was 208 mL, and the mean post-operative post-void residual was 150 mL, indicating an improvement of 58 mL in post-void residual scans, indicating an improvement in bladder emptying.

## Discussion

Urological care has been transformed by the advent of minimally invasive surgical techniques (MISTs), which provide efficient solutions with shorter recovery periods and fewer intra-operative and post-operative problems. Out of them, the UroLift treatment shows potential as a substitute for the conventional gold standard TURP. Despite its effectiveness, TURP carries some risks, including the possibility of post-operative haemorrhage (0.4%-7.1%) and, less frequently, problems such as erectile dysfunction and infertility [7]. Because of these concerns, safer alternatives must be investigated. By mechanically removing blockage without removing tissue, UroLift provides a novel method that maintains sexual function and speeds up recovery.

The findings of our study on the use of UroLift to manage BPH are consistent with outcomes reported in the existing literature, underscoring the potential of UroLift as a viable alternative to traditional surgical treatments such as TURP. In this discussion, we analyse the results in the context of other published data, highlight their clinical implications and address limitations for further exploration.

Efficacy of UroLift

The improvement in the median quality of life (QoL) scores and post-void residual (PVR) volumes observed in this study reflects the efficacy of UroLift in alleviating lower urinary tract symptoms (LUTS). Similar findings have been reported in multiple studies, with significant reductions in International Prostate Symptom Score (IPSS) and QoL scores, demonstrating the utility of UroLift in enhancing daily functioning and satisfaction [8,9]. The reduction in median PVR volume by 58 mL observed here aligns with other data suggesting improved bladder emptying after UroLift [6].

Safety profile and complications

The overall safety profile demonstrated in this study is favourable, with 67.18% (n=43) of patients experiencing no complications. Minor complications such as infections (6.25% (n=4)) and urinary retention (7.81% (n=5)) are consistent with the safety data reported in the literature [6,10]. Importantly, the absence of reported cases of retrograde ejaculation reinforces UroLift's reputation for preserving sexual function, as highlighted in prior studies [11]. The ability to perform UroLift under local anaesthesia or sedation broadens its applicability to older patients or those with comorbidities precluding general anaesthesia. In our study, 36% (n=23) of procedures were performed using alternatives to general anaesthesia, demonstrating its feasibility in diverse clinical settings. However, we observed no significant difference in complication rates or hospital stays based on the type of anaesthesia (p=0.8055 and p=0.1174, respectively), suggesting that its use should be guided by patient and provider preference. These findings align with previous studies demonstrating the adaptability of UroLift in outpatient and minimally invasive contexts [9].

Procedure failure

From this study, a failure rate of 17.18% (n=11), with 63.63% (n=6) of these cases requiring subsequent surgical intervention, falls within the 7%-22% failure range reported in the literature [6,10]. This emphasises how crucial it is to choose patients carefully. The variability in failure rates may depend on factors such as prostate size, the presence of a median lobe and the number of clips used [12]. Although the study's mean prostate size (48.63 mL) was within the NICE-recommended ranges, the results for larger prostates (>80 mL) are still unknown because of the paucity of available data.

Comparative advantage over TURP

Although TURP is still the gold standard surgical treatment for BPH, UroLift has several benefits, such as shorter recovery periods, sexual function preservation and the ability to be performed with minimal anaesthesia [11]. Although the majority of this group (64.06% (n=41)) underwent general anaesthesia, this may be due to institutional or patient preferences, However, using local anaesthesia reduces anaesthesia risks and broadens its use to patients with comorbidities [9].

Limitations and recommendations for future research

This study's retrospective design and single-centre scope limit generalisability. Careful patient selection must be observed in future studies as we found two patients out of the cohort who were not within the guidelines as set out by NICE: one patient aged less than 50 and one patient whose prostate size was more than 80 mL. Additionally, the two-year follow-up period is short for assessing long-term durability. Longitudinal studies with larger cohorts and multicentre data are essential for validating findings. Future research comparing UroLift with other minimally invasive techniques such as Rezum or HoLEP would enhance the evidence base. To improve the assessment of UroLift's safety and effectiveness, we suggest using the Clavien-Dindo scoring system in subsequent research to offer a consistent way to gauge the degree of complications.

Clinical implications

The findings support UroLift's status as a minimally invasive option with a good safety record for improving quality of life and relieving symptoms. It is especially appropriate for patients who are unsuitable for more invasive surgeries or who want to prevent sexual problems. Nonetheless, the comparatively elevated failure rate highlights the significance of thorough patient counselling and meticulous patient selection and anatomical assessment [6].

## Conclusions

This study reinforces UroLift's role as a minimally invasive treatment for BPH, particularly in the short to medium term. With appropriate patient selection, UroLift offers significant symptomatic relief, improved quality of life and a low complication rate. Future studies incorporating standardised complication grading and long-term outcomes will be crucial for optimising the procedure and refining selection criteria.
